# A JAK of All Trades: The Use of JAKi in a Patient With Erythema Annulare Centrifugum

**DOI:** 10.7759/cureus.88684

**Published:** 2025-07-24

**Authors:** Alison Romisher, Jordyn Kopelson, Sandy Milgraum

**Affiliations:** 1 Dermatology, Robert Wood Johnson University Hospital, New Brunswick, USA; 2 Dermatology, Cooper Medical School of Rowan University, Camden, USA

**Keywords:** erythema annulare centrifugum, figurate erythema, jak inhibitor, targeted immunotherapy, upadacitinib

## Abstract

Erythema annulare centrifugum (EAC) is a type of figurate erythema with an unclear etiology, often thought to be a delayed hypersensitivity reaction to various antigens. While many cases resolve spontaneously, chronic and refractory presentations can be challenging to manage. We report a case of EAC unresponsive to traditional treatments but demonstrating rapid improvement with upadacitinib, a selective Janus kinase (JAK) inhibitor. This case highlights a novel use for JAK inhibitors in treating EAC.

## Introduction

Erythema annulare centrifugum (EAC) is a figurate erythema characterized by slowly expanding annular or arcuate plaques. It is often idiopathic but may also be a reactive process triggered by infections, medications, or malignancies [1,2]. Unlike erythema gyratum repens, which expands rapidly, EAC expands a few millimeters per day [3]. EAC exists in two forms: superficial (with pruritus and trailing white scale) and deep (non-pruritic with a cord-like border) [1,2]. Treatment typically targets underlying triggers when identified. Otherwise, topical corticosteroids, calcineurin inhibitors, antifungals, or antibiotics can be used. Here, we report a refractory case of EAC that responded rapidly to upadacitinib, a selective JAK1 inhibitor.

## Case presentation

**Figure 1:**
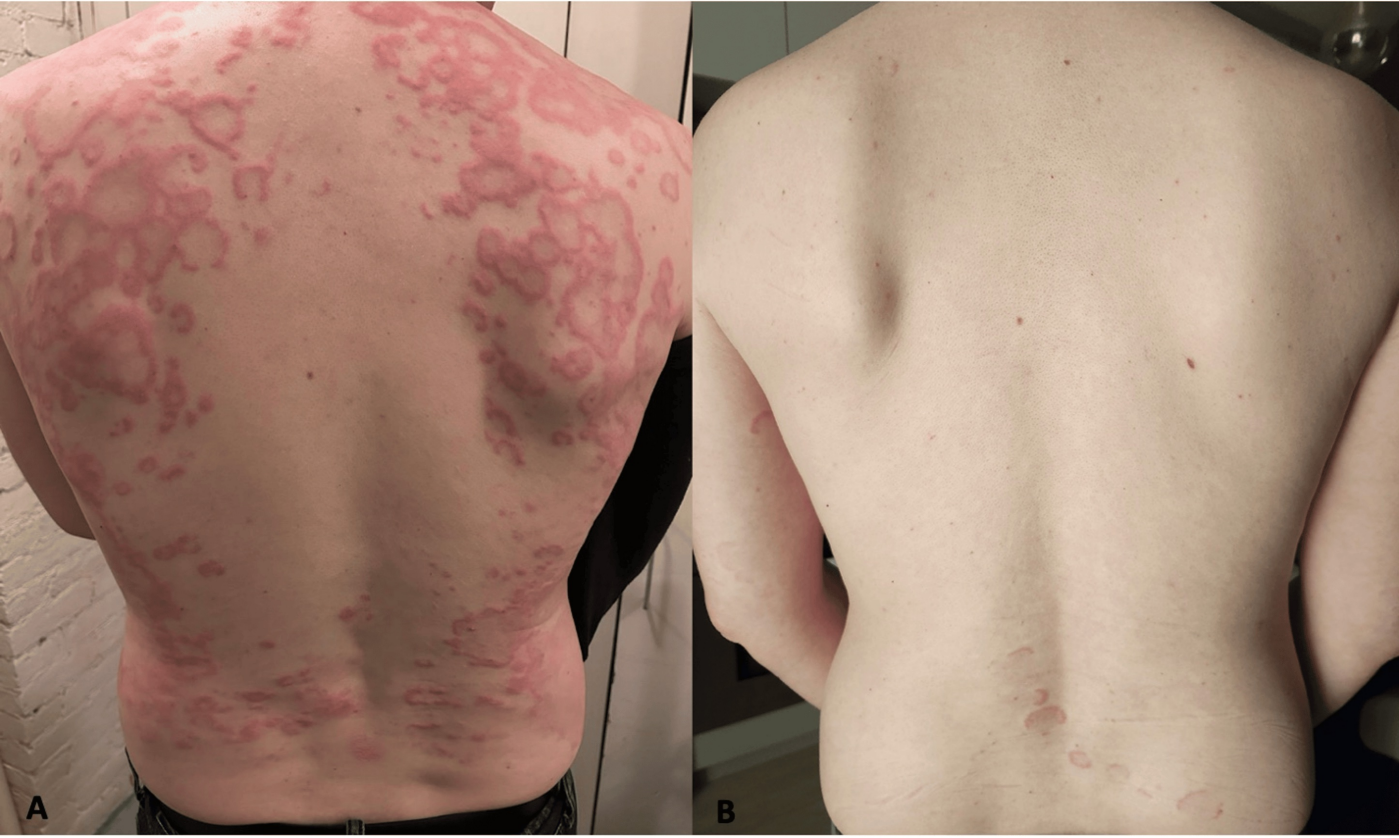
(A) At the initial visit, numerous arcuate and annular erythematous plaques with central clearing and cord like borders were observed; (B) After 12 weeks of upadactinib, the patient’s back was almost completely clear with few faded arcuate erythematous plaques on the lower mid back

**Figure 2:**
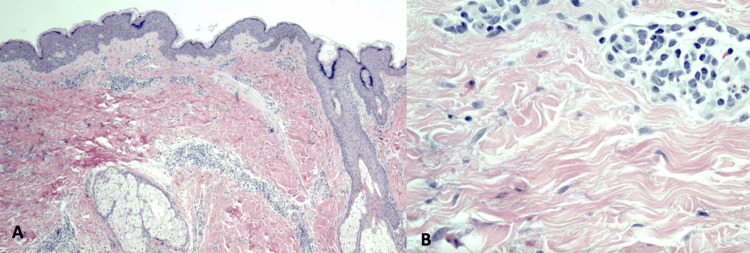
(A) Low magnification reveals minimal epidermal change. (B) High magnification reveals sparse, superficial and mid-dermal, perivascular, and interstitial lymphocytic infiltrates with scattered eosinophils and neutrophils.

A 37-year-old male with no significant medical history presented with the progressive eruption of annular plaques on the trunk, face, and extremities, starting two weeks after a 10-day course of amoxicillin-clavulanate for a chalazion. Lesions expanded 3-4 mm per day, ranging from 1-6 cm, lasting about two weeks before fading (Figure 1A). He denied systemic symptoms. Biopsy of a lesion revealed a superficial and mid-dermal perivascular lymphocytic infiltrate with scattered eosinophils and neutrophils (Figure 2). Clinicopathologic correlation was suggestive of EAC. An oral prednisone taper (40 mg x5 days, then 20 mg x5 days) yielded no improvement. A broad workup for infectious and autoimmune causes was unremarkable, including negative Lyme, rapid plasma reagin (RPR), antinuclear antibody (ANA), antineutrophil cytoplasmic antibodies (ANCA), and hepatitis panels, with mild C-reactive protein (CRP) elevation (7.5 mg/L) and normal erythrocyte sedimentation rate (ESR).

After an intramuscular triamcinolone injection (60 mg) also failed, the patient was started on upadacitinib (15 mg daily). Within two days, the lesions nearly resolved (Figure 1B). By six weeks, only a few new plaques appeared every two to three days. After 12 weeks, the rash resolved completely, though it recurred a few days after discontinuing therapy.

## Discussion

EAC is considered a hypersensitivity reaction, most commonly idiopathic but also associated with triggers like infections, malignancy (especially lymphoproliferative), medications, autoimmune disease, and pregnancy [3,4]. Reported drugs include penicillins, nonsteroidal anti-inflammatory drugs (NSAIDs), antimalarials, spironolactone, and rituximab [4]. Histologically, EAC resembles a pseudo-vasculitis, with tight perivascular lymphocytic infiltrates (the "coat-sleeve" pattern) in either the superficial or deeper dermis depending on subtype, but without the fibrinoid necrosis seen in vasculitis [3].

In this case, EAC likely developed secondary to recent antibiotic use. The eruption was resistant to both topical and systemic steroids but responded dramatically to the JAK1 inhibitor upadacitinib. This is, to our knowledge, the second reported case of EAC showing a response to JAK inhibition. In the previously reported case, the patient had failed mid- and high-potency topical corticosteroids, intramuscular triamcinolone, oral terbinafine, and dupilumab, but achieved complete clearance after 4 weeks of upadacitinib 15 mg daily [4].

JAK inhibitors, including upadacitinib, modulate cytokine pathways involved in various inflammatory dermatoses. Upadacitinib inhibits JAK1-dependent signaling from cytokines such as IL-4, IL-6, IL-13, and IFN-γ [5,6]. The patient’s rapid response suggests these pathways may play a key role in EAC’s pathogenesis.

Given their efficacy in other reactive dermatoses and chronic inflammatory skin conditions, including atopic dermatitis, psoriasis, and alopecia areata, upadacitinib and other JAK inhibitors may represent a promising therapeutic option in refractory cases of EAC. Further studies are needed to better elucidate the cytokine milieu in EAC and to define the potential role of targeted immunomodulatory therapies, such as JAK inhibitors, in its management.

## Conclusions

EAC can pose significant therapeutic challenges, particularly when unresponsive to conventional treatments. This case highlights that upadacitinib may represent a novel and promising alternative therapy for refractory cases of EAC. Additionally, this case may encourage broader consideration of the utility of JAK inhibitors in challenging dermatologic cases. As our understanding of cytokine signaling expands, JAK inhibitors may have applications well beyond what we currently imagine.
